# Lemierre’s syndrome caused by *Fusobacterium necrophorum* complicated with multiple brain abscesses—A case report, literature review, and suggested management

**DOI:** 10.1002/ccr3.5142

**Published:** 2021-12-04

**Authors:** Anders Kjellberg, Olof Bjerin, Elisabeth Franzén‐Röhl, Jiri Bartek, Peter Lindholm

**Affiliations:** ^1^ Department of Physiology and Pharmacology Karolinska Institutet Stockholm Sweden; ^2^ Hyperbaric Medicine Perioperative Medicine and Intensive Care Karolinska University Hospital Stockholm Sweden; ^3^ Neuropediatric unit The Institution for Women´s and Children´s Health Karolinska Institutet Stockholm Sweden; ^4^ Division of Infectious Diseases Department of Medicine Solna Karolinska Institutet Stockholm Sweden; ^5^ Department of Infectious Diseases Karolinska University Hospital Stockholm Sweden; ^6^ Department of Neurosurgery Karolinska University Hospital Stockholm Sweden; ^7^ Department of Clinical Neuroscience Karolinska Institutet Stockholm Sweden; ^8^ Department of Neurosurgery Rigshospitalet Copenhagen Denmark; ^9^ Division of hyperbaric medicine Department of Emergency Medicine School of Medicine University of California San Diego La Jolla California USA

**Keywords:** brain abscess, hyperbaric oxygen therapy, infectious diseases, lemierre syndrome, neurosurgery

## Abstract

We present an unusual case of Lemierre´s syndrome complicated by multiple brain abscesses, a literature review and suggested management. A young man with multiple brain abscesses deteriorated despite two weeks of directed antibiotics. A multidisciplinary approach was successful. Hyperbaric oxygen treatment (HBOT) should be considered in refractory or severe cases.

## Graphical abstract

Brain abscesses are rare. There are no well‐established international guidelines. Mortality and neurological sequelae rates are high. Hyperbaric oxygen treatment (HBOT) should be considered in refractory or severe cases.

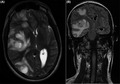

## 1 INTRODUCTION

Brain abscesses are rare, and currently, there are no well‐established international guidelines for treatment.^1^ Evidence is poor, and treatment recommendations are based on case series, retrospective studies, expert opinions, and personal preferences.^2^, ^3^ The incidence of brain abscesses is 0.3–0.9/100,000, and even though outcome has improved significantly over the past 50 years, mortality is still around 15% with neurological deficits in 30% of patients.^3^ Brain abscesses caused by *Fusobacterium Necrophorum* (*F*. *necrophorum)* are extremely rare but described in association with Lemierre's syndrome.^4^, ^5^ The treatment options include (1) empirical antibiotic therapy with cephalosporin and metronidazole, (2) surgical drainage for diagnosis and volumetric decompression, and (3) glucocorticoids to reduce edema.^6^ In the literature, a small case series with mixed pediatric^7^ and adult patients,^8^ and a recent case‐control study with 40 adult patients have reported on the positive effect of adjuvant hyperbaric oxygen treatment (HBOT).^9^ Here, we present a case with treatment‐refractory multiple brain abscesses that showed prompt improvement and change in clinical course, which coincided with the initiation of HBOT. “This case report follows the CARE guidelines”.^10^

## 2 CASE PRESENTATION

**FIGURE 1:**
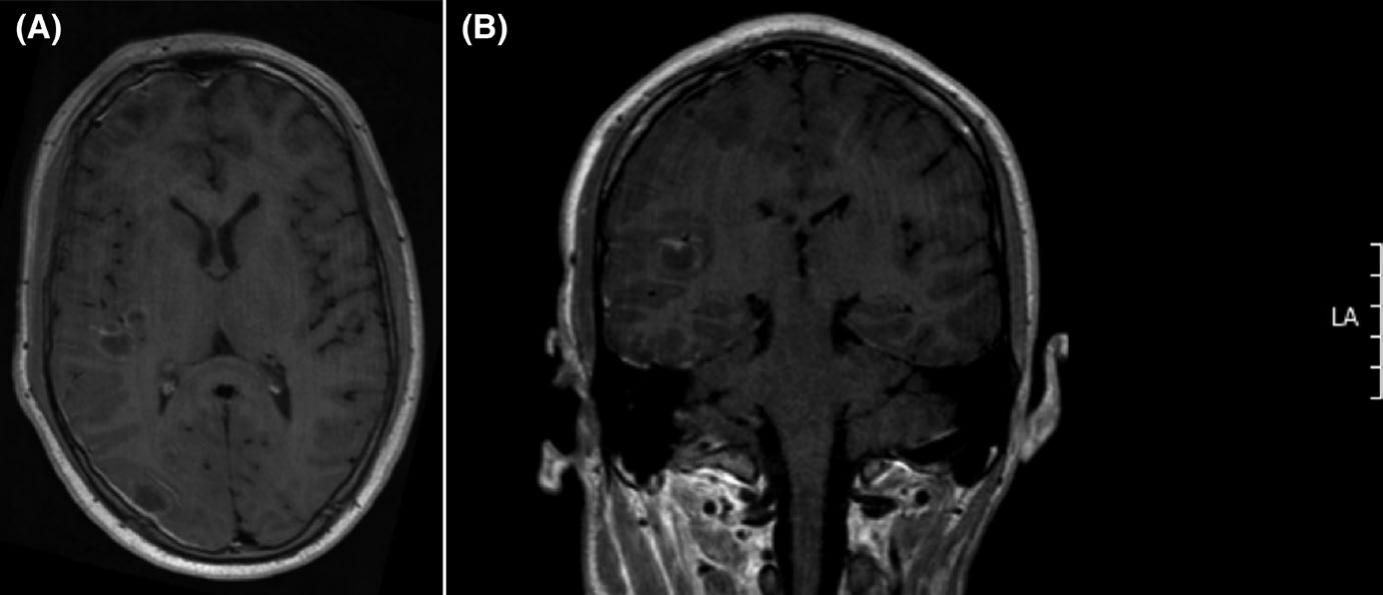
MRI Day two; axial (A) and coronal (B) T1‐weighted +contrast‐enhanced (CE) image demonstrating multiple intracranial abscesses

**FIGURE 2:**
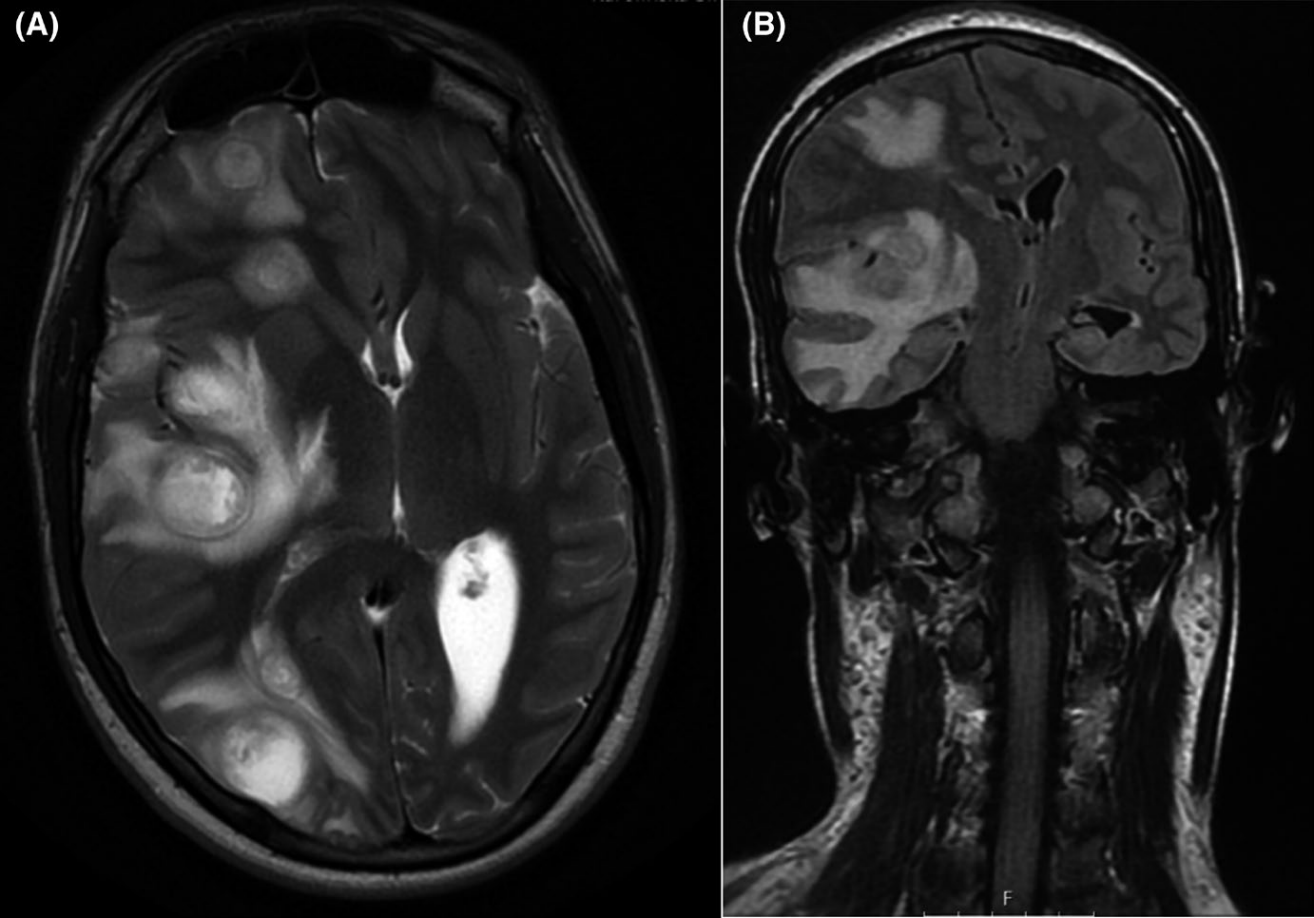
MRI Day two; axial (A) and coronal (B) T2‐weighted image demonstrating increasing edema and mass effect of intracranial abscess lesions

A 16‐year‐old, previously healthy, male patient presented initially to a general practitioner with neck pain and torticollis, which was interpreted as a benign condition, and he was sent home with paracetamol. The patient was then admitted to Karolinska University Hospital the day after when he presented again to the Emergency Department with sore throat, severe neck pain, and right‐sided Horner´s syndrome. A computed tomography (CT) scan of the brain showed a thrombosed internal carotid artery, a large abscess on the neck, and suspected multiple abscesses in the right hemisphere of the brain. A magnetic resonance imaging (MRI) scan the day after admission confirmed the suspected findings (Figures 1 and 2). The patient was started on intravenous antibiotics in the form of cefotaxime 2 g three times daily (TID) intravenously (4) in combination with clindamycin 600 mg TID iv plus metronidazole 1,500 mg once followed by 1,000 mg once daily (OD) iv, while the abscess on the neck was surgically drained. Furthermore, the patient was put on low‐molecular weight heparin 10.000 IU OD subcutaneously (sc) to prevent further thrombotic events.

The patient had multiple revisions of the neck abscess over the next few days, while the blood cultures came back positive for *F*.* necrophorum*, and antibiotics were changed to meropenem 2 g TID iv in combination with clindamycin 600 mg TID iv. A surgical intervention to drain the patient's multiple intracranial abscesses was deemed futile as: (1) diagnosis was already known and relevant antibiotic treatment initiated, and (2) the multiple abscesses were small in nature, without the possibility of significant volumetric reduction by drainage. Instead, based on previous experience^9^, ^11^ HBOT was suggested as a treatment adjunct, but not initiated since the patient showed slow improvement with reduced C‐reactive protein over the following 5 days. Nevertheless, no radiological decrease in the abscess formation was seen on an MRI performed at day 6. On Days 6–15, the patient gradually experienced a clinical decline with increasing pain; he was treated with high doses of opioids, clonidine, and eventually methadone (50 mg oxycodone, 17.4 mg methadone, and 300 µg clonidine daily). An MRI day 10 demonstrated increasing size of abscesses, increased edema (still moderate), and minimal midline shift. Betamethasone 4 mg daily was started on Day 13 (3 days before HBOT), and the dose was increased to 8 mg the day after the first HBO treatment. On Day 16, the patient's clinical condition worsened further with a decrease in consciousness to a Glascow Coma Scale (GCS) score of 13, a progressive left‐sided hemiparesis and severe pain. An MRI demonstrated increased edema, midline shift, and uncus herniation. The abscesses were mostly stationary in size, with some decreases or increases in comparison with an earlier (1 week previously) MRI exam. Once again surgery was deemed futile without the possibility of significant volumetric reduction by drainage. The patient was referred for HBOT treatment.

**FIGURE 3:**
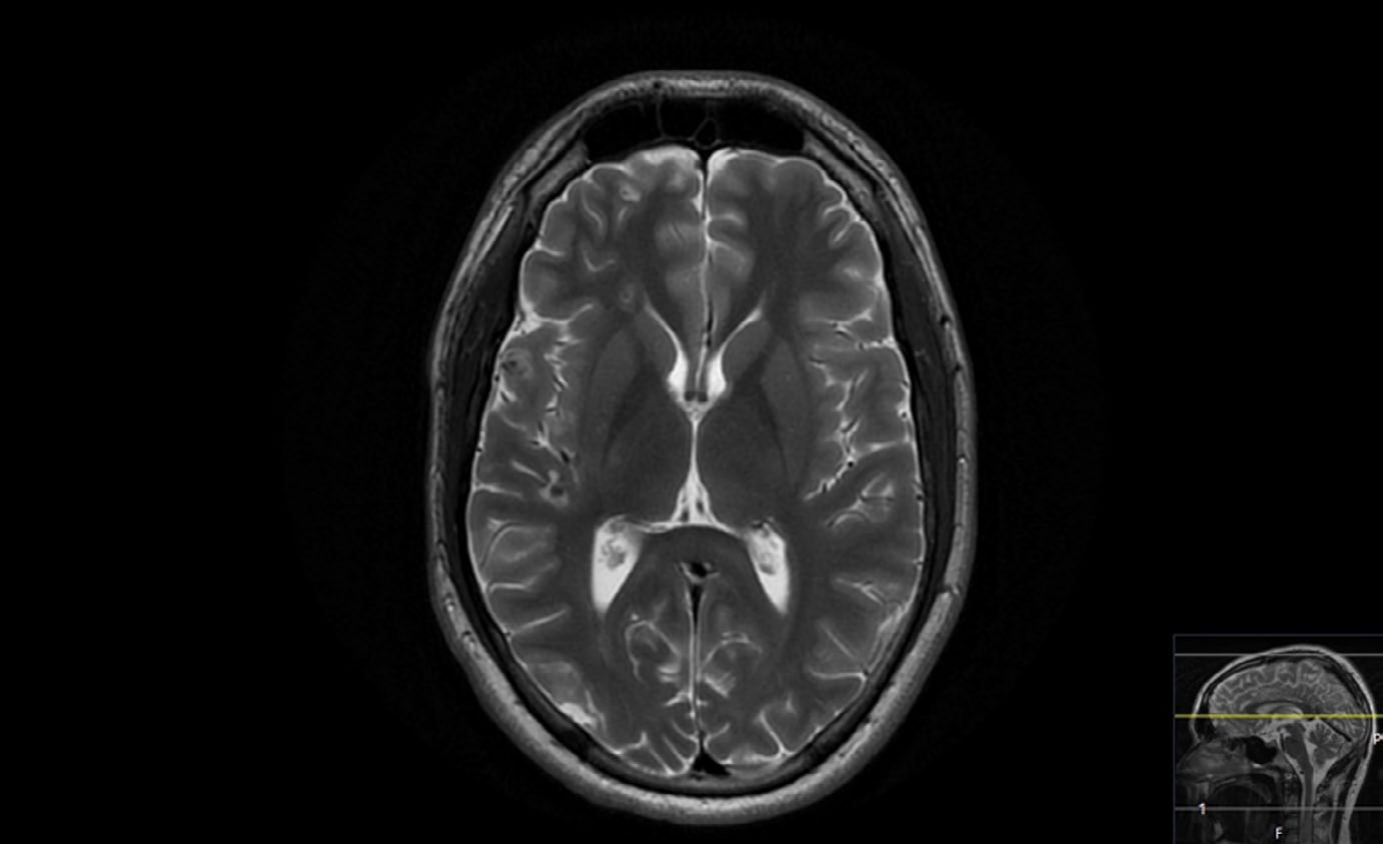
MRI at 7‐month follow‐up; axial T2‐weighted image demonstrating partial regression of perifocal edema; no new lesions; no abscess

**FIGURE 4:**
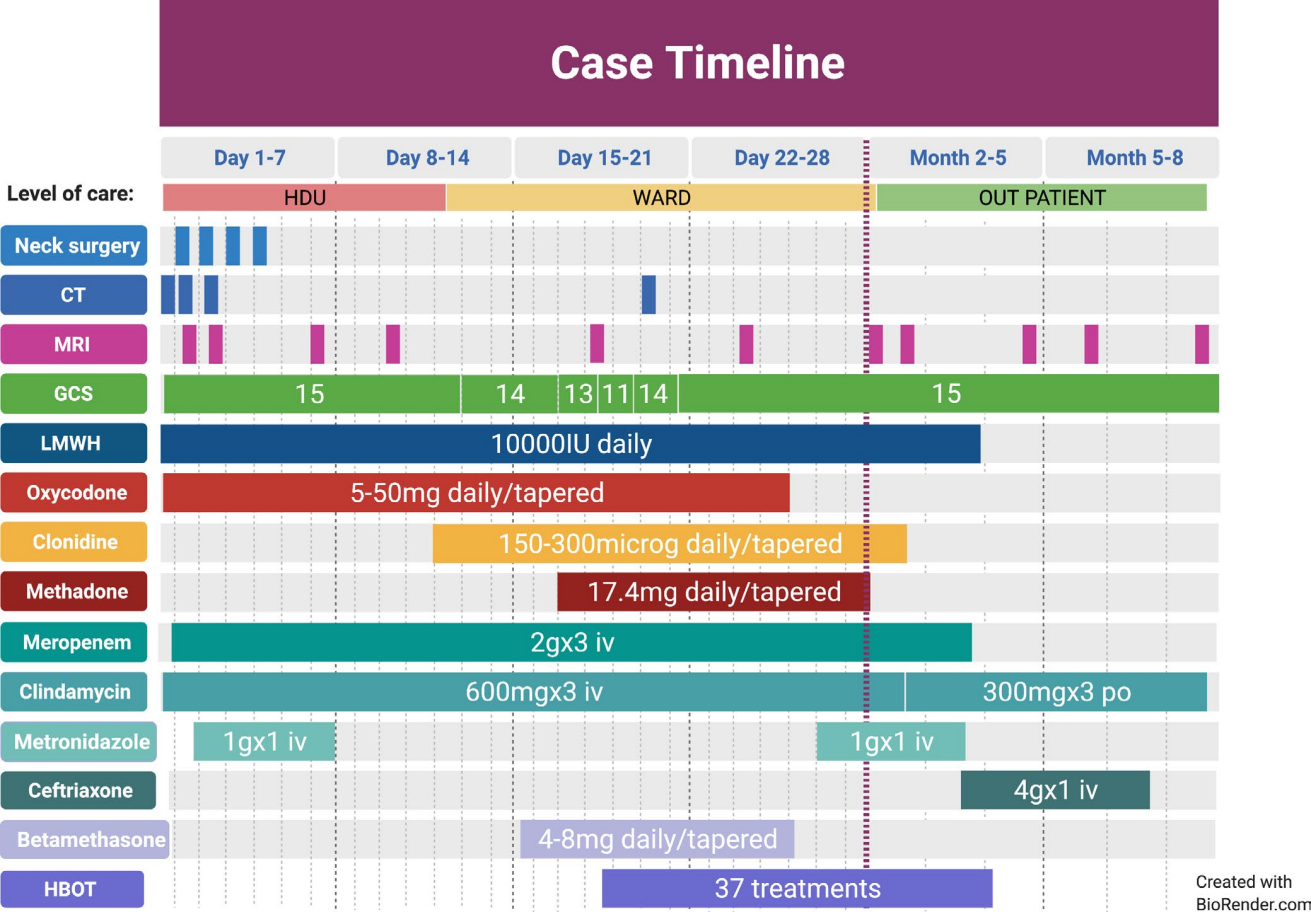
Case timeline

At HBOT initiation, the patient was afebrile and had a GCS of 11, severe pain, right fascial paresis, and paresis in the left arm. HBOT (280 kPa, 113 min in a multi‐place chamber) was initiated immediately upon referral, with the GCS increasing to 14–15 after the first HBOT session. Complete remission of the hemiparesis occurred after three HBOTs. All opioids were weaned after ten HBOTs. The patient was discharged to an out‐patients rehabilitation clinic after 15 HBOTs (received 37 HBOTs in total, no adverse events reported) and was continued on iv ceftriaxone 4 g OD iv for 2 months and long‐term oral clindamycin 300 mg TID po. He returned to school 2 months after presentation, feeling perfectly fine, why he did not want to complete the planned 40 HBO treatments. He went through neurorehabilitation and follow‐up as an out‐patient; neuro‐psychological assessment at 4 months after initial presentation was normal. MRI at 7 months showed complete remission of abscesses but a persistently occluded carotid artery and collateral flow to the right hemisphere (Figure 3). Antibiotics were discontinued at 8 months. The case timeline, including GCS, important diagnostic and medical interventions, and drug doses, is summarized in (Figure 4).

## 3 DISCUSSION

Brain abscesses are infrequent, and there are no international standardized consensus guidelines as to the use of antibiotics, surgery, or other adjunctive treatment(s). Attempts to establish consensus documents have been made, but there are still controversies regarding all forms of treatment, including type of empirical antibiotics and duration of treatment.^1^ There does seem to be a consensus that an initial CT‐scan should be followed by and MRI which is the gold standard for radiological diagnosis and to follow‐up on response to treatment.^12^ Empirically, a β‐lactam with good central nervous system‐penetration (such as a third‐generation cephalosporin in combination with metronidazole) is suggested for ear, nose, and throat origin or cryptogenic abscesses for 4–8 weeks.^2^ The timing and nature of potential surgical intervention (craniotomy or aspiration) have also been debated, and there is no conclusive evidence in favor of surgery, other than to secure a culture or as a rescue treatment to reduce increased intracranial pressure (volumetric reduction), which has been suggested if the medical condition is worsening despite administration of the correct antibiotics or if there are no clinical and radiological improvements within 1–2 weeks.^13^, ^14^ In the present case, neurosurgical intervention was deemed futile, since the possibility of volumetric reduction was minimal due to the multiple smaller abscess lesions that could not all be targeted for aspiration, while no single abscess represented enough volume to have any clinical significance.

The use of glucocorticoids as an adjunct treatment in these cases is controversial; no randomized studies are available. Glucocorticoids may negatively affect the state of the capsule and the penetration of antibiotics into the abscess. Glucocorticoids should be initiated in patients presenting with decreased consciousness, to reduce profound swelling and development of cerebral herniation. Treatment should be discontinued a couple of days after surgery and clinical stabilization. Glucocorticoids are not recommended as standard treatment, since it may prolong duration of antibiotic treatment but is often used to reduce cerebral edema and has not been shown to impact mortality.^15^

Hyperbaric oxygen treatment is not part of any guidelines for brain abscess treatment but the condition is one of 14 treatment indications recognized by the Undersea and Hyperbaric Medical Society (UHMS).^16^HBOT has been used for decades as an effective treatment in complicated infectious disease cases, by facilitating macrophage and neutrophil killing capacity in hypoxic environments such as abscesses,^17^, ^18^ and is known to potentiate the effect of some antibiotics.^19^ At our institution, we have positive recent clinical experience with treating both intracranial abscesses and infected intracerebral implants with HBOT.^9^, ^11^ The limited evidence in the literature (retrospective data) shows that HBOT is safe, reduces infection recurrence, reduces the need for reoperation, and may even positively affect overall outcome.^8^, ^9^ Despite some evidence for a synergic effect of HBOT and antibiotics, patients are not always referred to HBOT due to (1) the cumbersome nature of this treatment with patients often needing treatment daily for more than two weeks, (2) the high costs of treatment administration, and (3) lack of high‐grade evidence. In this specific case with multiple (more than 15) abscesses with known microbiology, the neurosurgeons suggested the use of HBOT at an early stage in the disease course, but the referral was delayed until there were radiological signs of herniation, and the patient had deteriorated severely.

As to the mechanism of HBOT effect in this case, it has been suggested that sudden deterioration in Fusobacterial infections is caused by neutrophil activation with increased inflammation rather than lack of infectious control.^20^ Both HBOT and glucocorticoids (dexamethasone) have been shown to reduce neutrophil activation in animal models,^21^, ^22^ and HBOT has been shown to reduce brain edema effectively and positively affect outcome in clinical trials of brain tumor patients^23^ and patients with traumatic brain injury.^24^ In severe brain abscess cases, factors associated with adverse outcome are GCS <9 and intraventricular rupture of brain abscess (IVROBA).^25^ Prodromal signs for IVROBA with a mortality of up to 90% include severe headache, signs of meningeal irritation, and rapidly deteriorating clinical condition within 10 days of meningeal symptoms.^26^ In retrospect, it is obvious that our patient was a severe case and had prodromal signs of IVROBA. Betamethasone 4mg daily was started 3 days before HBOT, the dose was increased to 8mg the day after first HBOT, and it cannot be ruled out that this treatment added to the remarkably positive effect of HBOT.

Since this is an unusual and heterogeneous disease, it is also important to carefully monitor the clinical and radiological changes and have frequent multidisciplinary conferences in cases that do not respond to culture‐directed antibiotics. Randomized controlled trials are warranted to conclude if corticosteroids and/or HBOT should have a place in standard of care. Nevertheless, based on current evidence, HBOT may have a positive impact on functional outcome and neither HBOT nor steroids have a negative impact on mortality; neither should be discarded as a treatment option.

## 4 CONCLUSION

A 16‐year‐old male patient with Lemierre's syndrome and brain abscesses suffering from severe pain and deteriorating clinical and radiological condition had a remarkably fast recovery 3 days after the initiation of HBOT. We can only infer an association with timing, not causality, but the improvement in clinical status did coincide with start of HBOT. There are some studies in the literature that support the use of HBOT in the treatment of cerebral abscesses, but the evidence is still weak, as for all treatment options for brain abscesses. This report highlights the need for consensus guidelines and multidisciplinary conferences when managing these patients. The use of HBOT should be considered in refractory or severe cases. Randomized controlled multicenter trials are warranted to establish high–grade evidence‐based guidelines, and to determine, if HBOT can be recommended as routine treatment in brain abscess management.

## CONFLICT OF INTEREST

The authors declare that they have no competing interests.

## AUTHOR CONTRIBUTIONS

AK drafted the manuscript together with PL. AK reviewed medical records and was involved in the case. OB reviewed medical records and was involved in the case. EFR contributed with references, information regarding infectious medicine management and was involved in the case. JB reviewed medical records and contributed with references and MRI pictures. AK, PL, OB, EFR, and JB contributed with critical review and amendments to the manuscript. All authors have read and approved the final manuscript.

## ETHICAL APPROVAL

The need for ethics approval was waived according to national regulations.

## CONSENT

Written informed consent was obtained from the patient's father and the patient for publication of this Case Report and accompanying images.

## Data Availability

Data sharing is not applicable to this article as no datasets were generated or analyzed during the current study.
